# Viral Gene Therapy for Glioblastoma Multiforme: A Promising Hope for the Current Dilemma

**DOI:** 10.3389/fonc.2021.678226

**Published:** 2021-05-13

**Authors:** Junsheng Li, Wen Wang, Jia Wang, Yong Cao, Shuo Wang, Jizong Zhao

**Affiliations:** ^1^ Department of Neurosurgery, Beijing Tiantan Hospital, Capital Medical University, Beijing, China; ^2^ China National Clinical Research Center for Neurological Diseases, Beijing, China; ^3^ Center of Stroke, Beijing Institute for Brain Disorders, Beijing, China; ^4^ Beijing Key Laboratory of Translational Medicine for Cerebrovascular Disease, Beijing, China; ^5^ Beijing Translational Engineering Center for 3D Printer in Clinical Neuroscience, Beijing, China; ^6^ Savaid Medical School, University of the Chinese Academy of Sciences, Beijing, China

**Keywords:** gene therapy, viral therapy, glioblastoma multiforme, viral vector, treatment strategy

## Abstract

Glioblastoma multiforme (GBM), as one of the most common malignant brain tumors, was limited in its treatment effectiveness with current options. Its invasive and infiltrative features led to tumor recurrence and poor prognosis. Effective treatment and survival improvement have always been a challenge. With the exploration of genetic mutations and molecular pathways in neuro-oncology, gene therapy is becoming a promising therapeutic approach. Therapeutic genes are delivered into target cells with viral vectors to act specific antitumor effects, which can be used in gene delivery, play an oncolysis effect, and induce host immune response. The application of engineering technology makes the virus vector used in genetics a more prospective future. Recent advances in viral gene therapy offer hope for treating brain tumors. In this review, we discuss the types and designs of viruses as well as their study progress and potential applications in the treatment of GBM. Although still under research, viral gene therapy is promising to be a new therapeutic approach for GBM treatment in the future.

## Introduction

Glioblastoma multiforme (GBM) is one of the most common primary brain tumors in adults (1, 2), mainly derived from astrocytes (3, 4). The World Health Organization (WHO) classification in 2016 defined GBM as grade IV, which leads to a high degree of malignancy and mortality. The current standard treatment for GBM includes maximum surgical resection, radiotherapy, and chemotherapy (5–7). However, the complete resection of GBM has been challenging due to the invasive growth pattern and the functional area involvement. It has been almost inevitable that the tumor-infiltrating parenchyma tissue eventually relapsed even after surgical resection (8, 9). The resistance to chemotherapy drug temozolomide (TMZ) was mainly caused by O6-methylguanine-DNA methyltransferase over-expression, mismatch repair and base excision repair (10–12). The molecules mediated GBM chemoresistance, including P-glycoprotein, multidrug-resistance protein transporters, and DNA repair enzymes (13). Furthermore, the inefficient delivery across the blood-brain barrier (BBB) limited the entry of therapeutic drugs into the central nervous system (CNS) (14–16). Glioblastoma stem cells supported tumor self-renewal which contributed to GBM resistance to radiotherapy (17–19). So even with standard treatment, the outcome of patients with GBM was still very poor. The median survival of patients diagnosed with GBM was just about 15 months (20–22). The clinical use of additional therapies, including local adjuvant therapy with Carmustine wafers and tumor-angiogenesis inhibition with Bevacizumab, were tried to improve the outcome (23–26). However, the survival rate was still less than 5% within five years of diagnosis (27, 28).

Therefore, the application of new treatment methods to get rid of the limitations of conventional treatment has been necessary. Growing evidence has proved that tumor is a kind of genetic disease (29, 30). With the exploration in the treatment of other diseases, viral gene therapy has brought hope for the treatment of GBM (31–33). Both wild-type viruses and engineered viruses could be used for viral gene therapy. Non-lytic viruses were used for gene therapy and lytic viruses exert anti-tumor effects by inducing tumor cell lysis (34). Moreover, the lytic viruses exposed GBM antigens to the host immune system which stimulated a specific immune response to tumor cells (35). The natural sensitivity of GBM cells to virus infection has made viral therapy a promising prospect. The viral vectors were designed according to the characteristic of target cells, the size of therapeutic gene, and the ability of long-term gene expression. In this review, we will focus on the types of viral vectors and demonstrate the versatility of gene therapy for GBM treatment. In this review, we will focus on the types of viral vectors (**Table 1**) and demonstrate the versatility of gene therapy for GBM treatment.

**Table 1:** Modifications and mechanisms of the viral vectors used for GBM gene therapy.

Viral vector	Agent	Modification	Mechanism
Retrovirus	HSV-tk	suicide gene therapy, thymidine kinase (TK) gene transfer	converting ganciclovir (GCV) into active form GCV triphosphate
	TOCA511	suicide gene therapy, cytosine deaminase (CD) gene transfer	converting 5-fluorocytosine (5-FC) into active antineoplastic 5-fluorouracil (5-FU)
Lentivirus	shRNA-lentivirus	sh-Bcl2 and S-TRAIL transfer	down-regulating Bcl-2 and inducing S-TRAIL expression
	sh-SirT1 lentivirus	sh-SirT1 transfer	silencing SirT1 in CD133+ cells to improve radiotherapeutic sensitivity
	miR-100 lentivirus	miR-100 transfer	regulating FGFR3 to inhibit tumor growth and increase sensitivity to chemotherapy
	GAS1-PT*EN lentivirus*	growth arrest specific 1 (GAS1) and phosphatase and tensin homolog (PTEN) gene transfer	expressing GAS1 and PTEN equally to perform superimposed anti-tumor effect
Adenovirus	ONYX-015	E1B gene deletion	replicating in p53 pathway altered tumor cells
	Delta-24	E1A gene partial deletion, Delta-24 RGD: Arg- Gly-Asp peptide sequence incorporation	replicating in Rb/p16 tumor suppressor pathway defective GBM cells Delta-24 RGD: expressing αv integrins to enhance infectivity
Herpes simplex virus	HSV1716	RL1 gene (γ34.5) loci deletion	targeting cells with defects in PKR pathway
	C134	RL1 gene (γ34.5) loci deletion, human cytomegalovirus IRS1 gene transfer	expressing IRS1 protein to enhance replication
	G207	RL1 (γ34.5) and UL39 gene deletion	inhibiting viral replication in non-dividing cells
	rQNestin34.5v.2	RL1 gene (γ34.5) loci deletion, RL1 gene under control of nestin promoter.	replicating in PKR-deficient, nestin-positive tumor cells
Oncolytic virus	Pelareorep (REOLYSIN)	wild-type reovirus	replicating specifically in Ras pathway activated tumor cells
	TG6002	ribonucleotide reductase genes deletion vaccinia, suicide gene therapy, FCU1 gene transfer	direct oncolysis effect and prodrug conversion
	H-1PV	wild-type parvovirus	clathrin-mediated endocytosis, DNA damage response, and cell-cycle arrest
	PVS-RIPO	poliovirus-rhinovirus chimera	restrict replicating in CD155-expressing tumor cells

## Viral Vectors

### Retrovirus

Because of the special biological characteristics, retrovirus vectors were first attempted in gene therapy for glioma. Replicating retroviral vectors were able to deliver the prodrug activator genes, which were also called suicide genes, into tumor cells and integrate into host genomes (36, 37). When a prodrug was given, the protein expressed by the gene could convert the non-toxic drug into a cytotoxic substance, which led to GBM cell death (38). Even as tumor cells escape the killing of cytotoxic drugs, they could also be used as an integrated retrovirus set and continue to play a role in the events of GBM recurrence (39). Therefore, this method was also known as “suicide gene therapy”. In the initial clinical trial, the therapeutic effect of retroviral vectors encoding herpes simplex virus thymidine kinase (HSV-tk) on malignant brain tumors was evaluated. The researchers found that HSV-tk could convert ganciclovir (GCV) into an active form of GCV triphosphate, which inhibited DNA replication and cell division in tumor cells, resulting in an anti-tumor effect (40). However, the results also indicated the limitations in transfection inefficiency of retroviral vectors was inefficient (41).

Retroviral vector TOCA511 has been used to deliver cytosine deaminase (CD) gene into tumor cells (42). CD enzyme converted the prodrug 5-fluorocytosine (5-FC) to active antineoplastic 5-fluorouracil (5-FU) which caused the death of tumor cells. The preclinical studies observed that TOCA511 did not lead to widespread or uncontrolled replication, which proved the safety of TOCA511 treatment. The safety and activity of TOCA511 were further supported by molecular analyses (43). Moreover, another study found that in addition to direct cytotoxic effects, TOCA511 could also be used as a radiosensitizing agent (44). TOCA511 could increase the intratumor concentration of 5-FU and induce T cell-mediated antitumor immunity (45, 46). TOCA 511 has been shown to be safe and provide a significant survival benefit in the clinical trial (47). Recent results from the Phase III clinical trial showed that TOCA511 treatment significantly improved survival in patients with two or more recurrences (48).

### Lentivirus

Lentiviruses belonged to the retroviridae family (49, 50). Exogenous genes or shRNAs could be effectively integrated into the genomes of dividing or non-dividing cells to achieve the effect of persistent expression of the target sequence (51). Compared with retroviral vectors, lentiviral vectors were more stable and less prone to insertion mutation. The active transportation of pre-integration complex through the nucleopore was the unique mechanism of lentiviral vectors (52). Researchers constructed a lentiviral vector expressing shRNA to downregulate Bcl-2 and S-TRAIL to induce apoptosis in glioma cells. The result showed that lentivirus-mediated apoptosis resulted in an increase in the expression of activated caspase-3 and caspase-7, which accelerated the apoptosis of tumor cells (53). The transfection of target genes by lentiviral vectors could improve sensitivity of GBM to radiotherapy. The CD133^+^ cells in GBM were resistant to radiotherapy. A study down-regulated the expression of sirtuin 1(SirT1) in CD133^+^ by a lentiviral vector expressing shRNA (sh-SirT1). The results showed that the silence of SirT1 significantly enhanced the inhibition of tumor growth by radiotherapy and improved the mean survival rate of GBM (54).

Specific miRNAs were proved to be associated with the increase of proliferation, invasiveness, angiogenesis, and apoptosis resistance in GBM. A study has shown that with the transfection of lentiviral vectors, the overexpression of miR-100 significantly inhibited the growth and migration of GBM, and increased the sensitivity to chemotherapy. And the delivered miR-100 played an anti-tumor effect on GBM by regulating FGFR3 directly (55). The latest genome editing technology could also be used for GBM treatment by lentiviral vector transfection. It has shown that editing the sequence of vascular laminin-411 overexpressed in GBM could suppress tumor growth and improve survival of GBM (56). Researchers constructed lentiviral vectors with an equal expression of growth arrest specific 1 (GAS1) and phosphatase and tensin homolog (PTEN) *via* the versatility of expression cassettes allocation. Both of the transgenes were regulated by the same promoter. The result showed that the anti-tumor effect of GAS1 could be superimposed with the inhibitory effect of PTEN on Akt pathway, and this could significantly inhibit the growth of GBM (57).

### Adenovirus

Adenovirus is a non-enveloped double-stranded DNA virus (58, 59). Adenoviruses selected for gene therapy were serotypes 2 and 5 (60). Adenoviral vectors used coxsackie-adenovirus receptor (CAR) to mediate cell tropism and internalize adenovirus vectors by the interactions between penton protein and host cell surface integrins (61–63). After endocytosis into the tumor cells, adenoviruses did not integrate into the host genome and remained episomal while gene expression (64).

The E1 and E3 regions of adenovirus genome were conventionally deleted to eliminate the expression-related toxicity by adenovirus infection. The latest generation of adenoviral vectors could minimize the anti-adenovirus immune response by removing all the endogenous virus coding regions to induce more stable transgene expression (65). The deficiency of non-replicative adenoviruses was that episomes might be diluted due to cell division, resulting in a rapid decline in transgene expression. Conditional replication adenoviruses, as tumor-specific agents, were designed to selectively replicate within and kill the tumor cells (66). Moreover, the replicated transgenes could spread the therapeutic effect to other neighboring tumor cells. The genetically modified adenovirus ONYX-015 was a recombinant chimeric Ad2 and Ad5 vector with selective replication ability. The protein encoded by adenovirus E1B gene interacted with tumor suppressor p53 and the transcriptional activity was blocked (67). Due to the decreased expression of p53 in GBM, ONYX-015 was able to replicate effectively. Previous studies showed that ONYX-015 administration was safe and effective (68). And the phase I clinical trial has shown that no serious adverse events were observed in patients treated with ONYX-015 and ONYX-015 therapy could significantly inhibit tumor growth (69).

Delta-24 was designed to selectively replicate in cells deficient in the Rb/p16 tumor suppressor pathway. The deletion of retinoblastoma (Rb) binding domain fragment in E1A gene inhibited the interaction between E1A and Rb. Rb protein negatively regulates cell growth by blocking E2F. Rb/p16 tumor suppressor pathway deficiency likely occurred in GBM cells, which made it possible for Delta-24 selective replication in GBM cells but not in the normal cells (70). Furthermore, it was difficult for adenoviral vectors transfection due to the poor expression of CAR in tumor cells, which reduced the therapeutic effect. By mortifying an Arg- Gly-Asp peptide sequence in the HI loop of the fiber, the vectors were allowed to bind αv integrins to enter the tumor cells, thus enhancing the infectivity of the virus (71). Delta-24 and its modified versions have shown encouraging results in clinical trials (72).

### Herpes Simplex Virus

Herpes simplex viral vectors used for gene therapy were mainly modified from Herpes Simplex virus type 1 (HSV-1), an enveloped double-stranded DNA virus (73). Due to the neurotropic nature, HSV vectors are attractive for gene transduction in central nervous system tumors. The 152kbp genome length made it possible to carry a sufficient payload (68).

RL1 gene (γ34.5) was a necessary gene for effective replication of HSV. RL1 gene encodes The Infected Cell Protein 34.5 (ICP34.5), also known as Neurovirulence factor ICP34.5, was encoded by RL1. Phosphorylation of translation initiation factor eIF2α by protein kinase R (PKR) inhibited the translation process and blocked the production of viral proteins. Moreover, PKR could activate transcription factor NF-κB by inducing the degradation of negative regulator IκB to stimulate the antiviral immune response. ICP34.5 reversed this process by activating phosphatase-1α (74). The PKR pathway was often inhibited in GBM, so it did not restrict the replication of HSV vectors with the modification of ICP 34.5. For example, the recombinant vector HSV1716 removed both copies of RL1 to allow its selective replication in tumor cells (75). Clinical studies have proved that HSV-1716 can effectively improve the survival of patients (76, 77). C134 vectors deleted RL1 gene and inserted human cytomegalovirus IRS1 gene to enhance replication (78).

Another important gene UL39 encoded the large subunit of ribonucleotide reductase (RR), also known as ICP6. This protein converts ribonucleotide into deoxyribonucleotide allowing viral DNA replication, and the UL39-deficient vectors were unable to replicate in non-dividing cells. However, the host ribonucleotide reductase could compensate for the function loss of viral RR in dividing cells. Combining the two mechanisms above, the deletion of RL1 gene in G207 allowed the virus to target GBM cells, and the mutation in UL39 gene eliminated the replication in normal non-dividing cells (79). The result of the clinical studies has shown the safety of G207 in GBM treatment, and the favorable therapeutic effect of the combination with G207 and radiotherapy in recurrent GBM treatment (80, 81). Due to the specific up-regulation of nestin promoter in gliomas, rQNestin34.5v.2 vectors were designed within an insertion with a copy of RL1 gene under the transcriptional control of nestin promoter. The combination of the vectors and cyclophosphamide was proved to increase virus replication in tumors and improve the survival rate of patients (82).

### Oncolytic Virus

Oncolytic viruses had dual anti-tumor effects, which not only destroyed tumor cells directly but activated tumor-specific immune response. Current clinical trials have demonstrated the feasibility of OV-specific tumor infection. By selective transfection and replication (83, 84), tumor cell lysis was induced without damage to normal cells. Furthermore, oncolytic viruses were also able to infect tumor vascular endothelial cells, inhibit tumor-related angiogenesis, and cause hypoxic death of tumor cells (85). Meanwhile, oncolytic viruses induced systemic anti-tumor immunity by releasing tumor-associated antigens (86).

In addition to wild-type viruses, engineered viruses could also be used as oncolytic viruses, including reovirus, vaccinia virus, parvovirus, poliovirus, vaccinia virus, Newcastle disease virus, etc. And the anti-tumor immunity could be further enhanced by encoding cytokines, chemokines, and tumor-associated antigens (87). Currently, types of viruses have been involved in clinical trials to verify the safety and therapeutic effectiveness. Reovirus was a non-enveloped wild-type oncolytic virus with double-stranded RNA genome. Reovirus Pelareorep (REOLYSIN) could replicate specifically in Ras pathway activated tumor cells (88). In the phase I clinical trial, no treatment-related adverse reactions after intratumoral injection of reovirus were observed (89). Reovirus therapy could lead to tumor leukocyte infiltration and an increase in the expression of IFN, caspase 3, and PD-L1 (90). As an enveloped double-stranded DNA virus, the vaccinia virus did not rely on cell receptors, but membrane fusion to enter cells. Its rapid replication cycle and strong ability of intercellular transmission made it a promising candidate for viral therapy (91). TG6002 was modified from vaccinia virus, which was designed as a combination of direct oncolysis effect and prodrug conversion function. It mainly replicated in tumor cells and transformed 5-FC into 5-FU. Its safety and oncolytic activity have been confirmed in a large number of preclinical studies (68). Parvovirus was a single-stranded DNA virus. As a kind of parvovirus, H-1PV bound to the receptors on the surface of host cells and entered within endocytosis mediated by clathrin, inducing DNA damage and cell cycle arrest (92). The result of clinical trials showed that H-1PV could cross the BBB to reach the tumor and enhance the immunogenicity in tumor microenvironment (93, 94). Poliovirus was a kind of encapsidated viruses with a single strand RNA. Poliovirus infected tumor cells by binding the cell adhesion molecule CD-155 expressed in GBM (95). The phase I clinical trial showed that PVS-RIPO (poliovirus-rhinovirus chimera) immunotherapy significantly improved the survival rate of GBM patients (96).

## Gene Therapy

Gene therapy achieved the purpose of treatment by delivering therapeutic genes or manipulating disease-related genes into target cells. Based on the related therapeutic strategies, gene therapy has been divided into suicide gene therapy, oncolytic viral gene therapy, tumor suppressor gene therapy, immuno-stimulatory therapy, and tumor microenvironmental regulation therapy (97, 98). Suicide gene therapy and oncolytic viral gene therapy have been described above. The main function of tumor suppressor genes included cell signal transduction and epigenetic regulation, negative regulation of cell cycle, negative regulator expression, regulation related to stem cell proliferation, and DNA mismatch repair. Studies have shown that Rb, p53, PTEN, CDKN2A, and other tumor suppressor genes played an important role in effective GBM inhibition. However, the related clinical trials on tumor suppressor gene therapy were limited. IFN-β (interferon β) inhibited the growth and invasion of GBM with the effects of anti-tumor immune modulation, anti-proliferation, and anti-angiogenesis (99). IFN-β gene delivered by viral vectors showed a widespread expression and distribution in astrocytes and endothelial cells. A phase I clinical trial showed local inflammation and tumor necrosis in IFN-β treatment (100). The intra-tumor injection of angiostatin could effectively inhibit tumor growth and vascularization (101, 102). Therefore, anti-angiogenic genes and tumor extracellular matrix regulatory genes made it possible to treat GBM *via* modulating tumor microenvironment.

## Discussion

Glioblastoma has been a common, highly aggressive, and heterogeneous brain tumor. The infiltration of GBM to the surrounding tissue made it impossible to eliminate by surgical intervention. The inefficient delivery of BBB reduced the therapeutic effect of chemotherapy. The abnormal vascularization promoted the proliferation of tumor cells. And immunosuppressive status in tumor microenvironment severely limited the anti-tumor response to GBM. Therefore, we urgently need new treatment strategies to face the challenges of this disease and improve the prognosis of patients. Gene therapy aimed to treat GBM by targeting and regulating oncogenes and tumor suppressor genes in tumor cells. The latest understanding of genetic material and molecular alteration provided an accurate theoretical basis for gene therapy. Due to the high transfection efficiency and the development of vector engineering techniques, viruses were widely used in the researches of GBM gene therapy. Viral vectors-mediated gene therapy could be combined with current treatment methods to improve therapeutic outcomes. The transmission of suicide genes has been evaluated in clinical trials to overcome the resistance of chemotherapy. HSV-tk and TOCA511 converted the prodrugs into active form and mediate the anti-tumor response. The transfection of sh-siRT1 vectors in CD133+ GBM cells significantly improved the resistance to radiotherapy. The extensive replication of oncolytic viruses in tumor cells, including ONYX-015, Delta-24, and PVS-RIPO, led to cytolysis and induced an anti-tumor immune response. Furthermore, the expression of cytokines could enhance the therapeutic effectiveness of viral vectors by improving anti-tumor immunity. However, there are still some concerns that need to resolve in viral gene therapy before its application in clinical therapy. The first is the transduction efficiency and expression stability of target genes. As we mentioned, the expression level of receptors and the efficiency of membrane fusion affected the entry of the virus into tumor cells. How well the viruses entered the cells would determine the effectiveness of gene therapy. Non-replicative viruses, including adenoviral vectors, were not integrated into the host genome, the expression level of the transgenes might decrease with cell divisions. Secondly, viral vectors needed to be further optimized to improve tumor targeting especially in radiotherapy and chemotherapy-resistant GBM cells, and avoid entering normal cells. Multiforme implied that heterogeneity existed among the GBM cells within the same tumor. It has been necessary to explore the common mechanism of viral replication. Moreover, the use of engineering technology to eliminate the immunogenicity of virus was worth considering, which could avoid antiviral immunity. Currently, several preclinical trials and clinical trials have proved the safety of viral therapy. However, the effectiveness of its treatment in clinical trials was still unclear, so large clinical trials have been needed. Undeniably, viral gene therapy provided a new therapeutic approach and perspective in GBM treatment.

## Conclusion

Viral gene therapy has shown strong therapeutic potential in GBM treatment. In the future, studies need to focus on the therapeutic efficacy and monitor adverse events before viral vectors widely use in clinical practice. Furthermore, the combination of viral gene therapy with other new treatment methods needs further research. Although the road ahead may be challenging, gene therapy has brought new hope for patients with GBM.

## Author Contributions

All authors listed have made a substantial, direct, and intellectual contribution to the work, and approved it for publication.

## Conflict of Interest

The authors declare that the research was conducted in the absence of any commercial or financial relationships that could be construed as a potential conflict of interest.
